# Ecosystem services from southern African woodlands and their future under global change

**DOI:** 10.1098/rstb.2015.0312

**Published:** 2016-09-19

**Authors:** Casey M. Ryan, Rose Pritchard, Iain McNicol, Matthew Owen, Janet A. Fisher, Caroline Lehmann

**Affiliations:** 1 School of GeoSciences, University of Edinburgh, Edinburgh EH9 3FF, UK; 2 Kikenni Consulting, Barn Cottage, Moorland Street, Axbridge BS26 2BA, UK

**Keywords:** global change, miombo, mopane, land use, energy, woodfuels

## Abstract

Miombo and mopane woodlands are the dominant land cover in southern Africa. Ecosystem services from these woodlands support the livelihoods of 100 M rural people and 50 M urban dwellers, and others beyond the region. Provisioning services contribute $9 ± 2 billion yr^−1^ to rural livelihoods; 76% of energy used in the region is derived from woodlands; and traded woodfuels have an annual value of $780 M. Woodlands support much of the region's agriculture through transfers of nutrients to fields and shifting cultivation. Woodlands store 18–24 PgC carbon, and harbour a unique and diverse flora and fauna that provides spiritual succour and attracts tourists. Longstanding processes that will impact service provision are the expansion of croplands (0.1 M km^2^; 2000–2014), harvesting of woodfuels (93 M tonnes yr^−1^) and changing access arrangements. Novel, exogenous changes include large-scale land acquisitions (0.07 M km^2^; 2000–2015), climate change and rising CO_2_. The net ecological response to these changes is poorly constrained, as they act in different directions, and differentially on trees and grasses, leading to uncertainty in future service provision. Land-use change and socio-political dynamics are likely to be dominant forces of change in the short term, but important land-use dynamics remain unquantified.

This article is part of the themed issue ‘Tropical grassy biomes: linking ecology, human use and conservation’.

## 1. Introduction

**Figure 1.**
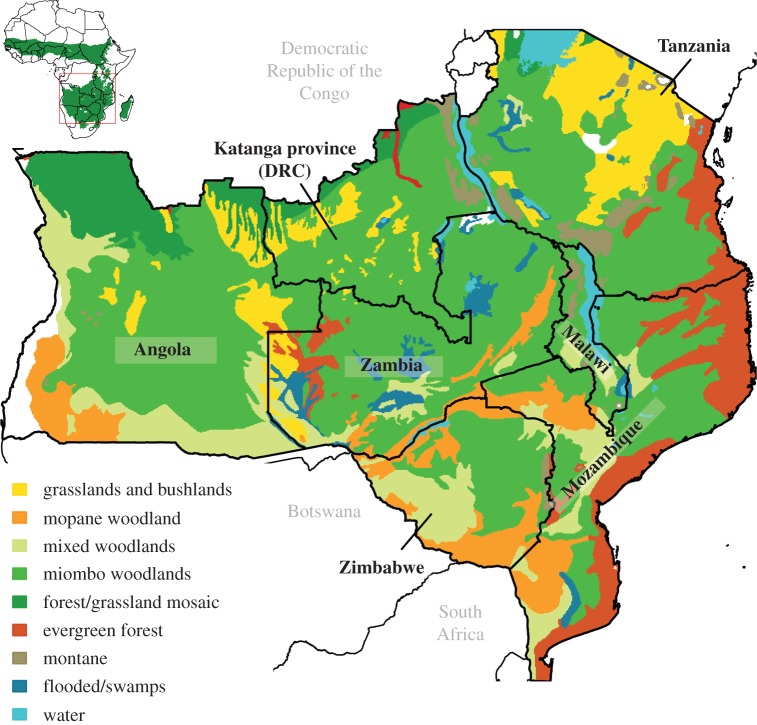
The potential extent of the miombo and mopane woodlands of southern Africa, based on vegetation maps and expert opinion [2]. Conversions to croplands, urban areas etc. are not indicated. The inset shows the extent of African savannas [3].

The ‘social woodlands’ of southern Africa are the dominant land cover of the sub-continent, and consist of woody savannahs dominated by trees of the Caesalpinioideae [1] (figure 1). Ecosystem services (ES) from these woodlands provide important contributions to the livelihoods of over 100 M rural people and 50 M urban dwellers, mitigating some of the symptoms of the chronic poverty in the region [4]. Woodland ES include a hyper-diverse range of provisioning services used by rural households (e.g. fuelwood, building materials and fruit), urban consumers in the region (e.g. charcoal, bushmeat and medicines) and internationally [1,5]. Supporting and regulating services provided by woodlands include the cycling of nutrients, which is important to the region's agriculture, carbon cycling, and perhaps the regulation of water and soil movements. Myriad cultural services are provided by woodlands, including tourism and spiritual associations. Miombo (dominated by trees of the genera *Brachystegia* or *Julbernardia*) and mopane (*Colophospermum mopane*) woodlands are the main components of a ‘Zambezian’ region of Africa, distinct in terms of mammals, birds, amphibians, reptiles and plants, with high levels of diversity and endemism [6,7].

A wealth of literature attests to the importance of ES from these woodlands to the livelihoods of the poor [8], but questions remain about how this relationship will be altered by environmental and social changes [9]. Rapid changes in land use in the woodlands are anticipated, as the historical isolation from global markets and capital is reduced and the region integrated into the global land-use system [4,10]; the woodlands are thus increasingly seen as the last agricultural frontier in an era of land scarcity [11]. Meanwhile, climatic, atmospheric and other environmental change may alter the growth rates of woodland flora, impacting species composition and productivity. These changes will result in trade-offs and conflicts between the beneficiaries of different ES, some of which may feedback on the drivers of change. It is the impact of these changes (hereafter ‘global change’) on the flow of ES that is the subject of this paper.

Recent debates surrounding proposals for large-scale tree planting in grassy ecosystems [12] and our cultural neglect of savannahs [13] have highlighted deep miscommunications and probably misconceptions about the ES provided by tropical grassy biomes, and in particular the woodlands of southern Africa. Examining the ecological basis of the services provided by woodlands can help move this debate forward, by clarifying which services are likely to benefit from increased tree cover, and which may decline. In this paper, we first synthesize what is known about the ES provided by miombo and mopane woodlands and then assess how social and environmental change may alter these services in the future.

## 2. Material and methods

Following the Millennium Ecosystem Assessment, we define ES in purely anthropocentric, instrumental terms, in contrast with more ecological definitions often used by early socio-ecologists [14] and many ecologists. Our approach is also guided by a conceptual framework conceived within the Ecosystem Services and Poverty Alleviation programme [15]. This moves beyond the classical ‘cascade diagram’ of Kumar [16]—whereby ecosystem structure is linked to ecosystem function, to ES and to human well-being—to pay attention to the social factors that restrict access and provide control over ES [17]. Thus, we analyse not only the aggregate provision of ES but also, where possible, identify the social groups that benefit, and the capitals, assets and social-relational access mechanisms [18] that allow them to do so. We focus on the miombo and mopane woodlands themselves, and not all of the broader landscape of which they are a part. To maintain a semblance of holism, where possible we evaluate the connections between the woodlands and associated land covers, particularly agriculture and wetlands (*dambos*). The countries dominated by miombo and mopane considered in this paper are Angola, Zambia, Zimbabwe, Mozambique, Malawi and Tanzania. Although there are many other land covers in these countries, and woodlands are found outwith them, we are by necessity sometimes forced to use these boundaries as a proxy for the study area where only national datasets are available. This analysis has the advantage of building on previous excellent reviews which are used as a starting point throughout [1,4,5,19].

## 3. Provisioning services from woodlands

### (a) Wild foods and construction materials

Woodlands and associated wild land covers provide a very wide range of provisioning services that are used by local people, by nearby urban dwellers and internationally (table 1 and electronic supplementary material, table S1). This ‘hidden harvest’, absent from national accounts, includes woodfuel (dealt with separately below), wild foods, medicine and materials for construction. These have substantial economic value, accounting for around 26% of cash and subsistence income in rural areas. In absolute terms, this averages $518 ± 108 hh^−1^ yr^−1^ or $9 ± 2 billion yr^−1^ (electronic supplementary material, table S2) when scaled up to the regional rural population of 100 M people [20]. One important caveat is that non-wooded land including drainage lines, termitaria and field margins is often the source of these wild products. This is often overlooked in the classification of such products as non-timber *forest* products [21,22].

**Table 1.** A summary of the main provisioning services from woodlands, with an estimated ranking of their importance to a range of beneficiaries. A fully referenced table with notes and examples appears in the electronic supplementary material, table S1, and the economic value of such products in detailed in the electronic supplementary material, table S2.

	beneficiary				
product	local use as a safety net	local subsistence consumption	rural markets	urban/regional markets	international
wild foods					
wild fruits	high	high	medium	medium	medium
wild vegetables	medium	medium	low	no reports	no reports
mushrooms	low	medium	medium	low	no reports
edible insects	medium	medium	medium	medium	low
honey	low	low	medium	medium	low but increasing
bushmeat	medium	high	medium	medium	low
building and craft materials					
barks and fibres	low	medium	medium	medium	no reports
thatching grass	medium	high	high	medium	no reports
construction poles	low	high	medium	low	no reports
medicinal plants	low	high	high	high	medium

The beneficiaries of provisioning services vary widely, depending on the degree of commercialization [23]. Women are disproportionately involved in the harvesting, processing and sometimes consumption of many of these goods, although this changes in favour of men for labour-intensive commercial products such as charcoal, honey and timber [24,25]. Marginalized groups unable to compete in local labour markets depend heavily on these goods, and wild food nutrition is important for children [26]. Only a few products have international commodity chains (e.g. honey, marula and baobab fruit pulp and seed oil), despite considerable potential. Many case studies in the study region attest to the importance of these food sources during droughts or other household income shocks [25,27–29]. For instance, during a year with poor harvests, wild foods can account for 30% of calorie consumption [30]. However, a recent global analysis has questioned the prevalence of this coping strategy, indicating that households prioritize reducing consumption and selling assets in times of crisis and that wild land products play a minor complimentary role in the coping strategy portfolio [31].

### (b) Fuel

Woodlands provide 76% of total energy use in the region in the form of potentially renewable biomass. Total employment in the traded woodfuels sector is between 1.4 and 2.5 M people with a traded value of $780 M per year (table 2). Most woodfuel used in rural areas is in the form of wood and is collected locally, often harvested from already dead material, although this can change in situations of scarcity [39]. By contrast, urban people mainly consume traded woodfuels, primarily charcoal. These traded woodfuels are used by 70–90% of the 50 M urban population in the region [34,40,41] and support a significant flow of money from urban to rural areas (the higher values of woodland income in the electronic supplementary material, table S2 are typically in areas producing charcoal). The main beneficiaries are urban consumers who benefit from reliable, consistently priced energy [42], useable without substantial capital investment, and available in small quantities; rural producers who sell charcoal for 12–53% of the final price [36,41,43,44]; governments and their officials who tax woodfuel transport, most often privately (i.e. bribes) [45,46]; and wholesalers and retailers—for instance, charcoal retailing provides employment for poor urban women [47].

**Table 2.** Woodfuel consumption estimates from six countries dominated by miombo and mopane woodlands. Energy use data are from the International Energy Agency online database, except for Malawi which are based on Owen *et al*. [32]. Consumption figures are from Bailis *et al.* [33] with charcoal consumption converted to wood consumption using a factor of 6.1 [34]. Regional employment is estimated assuming that charcoal creates 200–350 job-days per terajoule of energy consumed and 260 work days per year [35]. Note that it is notoriously difficult to estimate the scale of employment in informal industries, and there are few reliable studies relating to the woodfuels sector. Blanks indicate where data are not available.

	biomass energy as % of total energy consumption, 2000	biomass energy as % of total energy consumption, 2012	fuelwood consumption (k tonnes yr^−1^)	charcoal consumption (k tonnes of wood equivalent yr^−1^)	total consumption of wood (k tonnes yr^−1^)	value of traded woodfuels	employment in traded woodfuels	notes
Angola	72	54	4179	6867	11 046			
Malawi	93	87	3173	2925	6098	3.5% of GDP	133 000 full time	collected wood valued as 1.6% of GDP, in addition to traded woodfuels. Value and employment figures from [32]
Mozambique	89	76	9543	5741	15 284	2.2% of GDP	214 000 (charcoal only)	value and employment data from [36]
Tanzania	92	87	26 962	9535	36 497	2.3% of GDP (Dar es Salaam only)	1 900 000 people-years	value and employment data from [37]
Zambia	79	78	7878	6089	13 967		500 000 (charcoal only) [38]	
Zimbabwe	64	71	10 525	31	10 556			
Woodland region	81	76	62 260	31 188	93 448	$780 M	1.4–2.5 M	see text for employment data

### (c) Other provisioning services

Commercial timber harvesting is an important provisioning service for both international and domestic consumers [48], with exports of wood products from the region being worth about $166 M per year (2011–2015, except Angola where data covers only 2004–2006) based on international trade statistics [49]. However, these data are highly unreliable due to likely illegality and other problems [50], and this service is not considered here in any more detail. However, it is important both as a mass-produced commodity and also for niche markets involving high-value timber [51].

## 4. Services supporting agriculture

**Figure 2.**
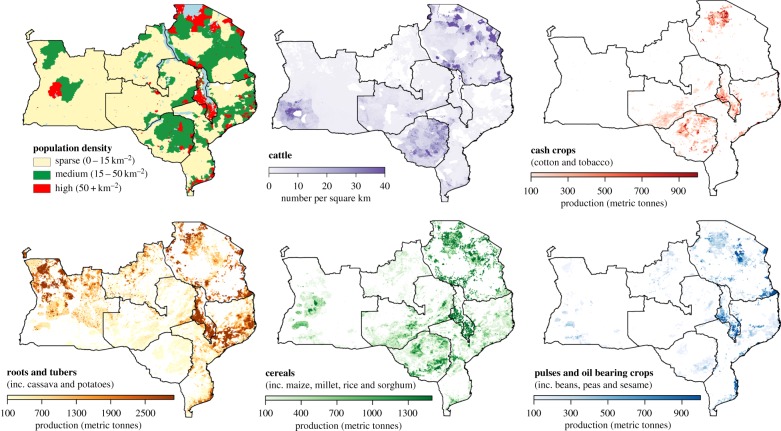
Human population density in 2000 (GRUMP v1 [59,60]), crop production in 2005 (MAPSPAM v2 [61]) and cattle in 2005 (Gridded Livestock of the World 2 [62]) in the woodland region. MAPSPAM data show the quantity of crop(s) produced in each 10 km by 10 km grid cell. The former Katanga province of the D. R. Congo is included in these maps as it is largely covered by woodlands; however, due to sparse subnational data it is not included in analyses elsewhere in this paper.

There are close linkages between agriculture and services provided by woodlands. The two key linkages are the ES of nutrient cycling and soil erosion regulation (although pollination services and pest regulation may be important in some cases, they are not explored here). Woodlands make nutrients available to crop production through woodland-field lateral transfers and through shifting cultivation. In the former, nutrients are transported to the field to boost yields [52] and sustain production on inherently infertile soils, reducing the need for chemical fertilizers and fallow periods. Common in Zimbabwe [52–56], this largely takes the form of the transport of woodland termitaria soils or leaf litter to the fields (often mixed with manure before application). More substantial in terms of nutrient mass flow is the addition to fields of manure from cattle grazed in woodlands. In Zimbabwe, it is estimated that most of the fodder for cattle comes from woodlands [54]—both as grass and, especially in the dry season, browse [57]. Nutrient additions from cattle manure are more common, and larger, among better-off farmers, who have more cattle [56] and transport equipment [53]; it also requires access to large areas of grazing land [58]. This service is only important in regions of semi-permanent agriculture where tsetse fly does not preclude livestock raising (figure 2).

Bringing the farm to the nutrients, though the process of shifting cultivation, is the other way in which woodland nutrient cycling supports crop production [63,64]. This practice is generally thought to be widespread in areas with population densities below 15 people km^−2^ [65], and if so, this would imply it is undertaken by 18% of the rural population (figure 2). Under a tree-fallow system, nutrients accumulated in the soil and plants are made available to crops by cutting and burning the phytomass of the nascent field, sometimes amended with branches from elsewhere [63]. Burning improves the pH of the soil, makes phosphorus available and increases available nitrogen through the addition of fine organic material and the suppression of nitrification [66].

Thus, overall it is clear that woodland nutrient cycling supports a large amount of the agricultural production in the region, mainly as a result of the limited use of mineral fertilizer [67]. This contribution is rarely counted in large-scale nutrient balances because of mismatches in temporal and spatial scale [68], so it is currently impossible to quantify its importance, but is clearly vital to the livelihoods of most of the region's farmers. The ultimate sources of these nutrient inputs are N fixation in the woodlands, soil formation and N deposition. These are all little studied in the region, and the relative importance of these processes is a key area for future research.

### (a) Regulation of soil erosion

Woodlands also support agriculture by keeping the relatively nutrient- and organic-rich surface soil in place, making it available for agricultural use at some future date. This is achieved through the interception of high-energy rain drops and by the structural integrity the vegetation gives the soil. Observed plot-level soil loss rates are around 0.4–1 t ha^−1^ yr^−1^ under woodland [69,70]. Rates are higher under agriculture: 2.5 ± 1.0 t ha^−1^ yr^−1^ in areas with small-scale cultivation; 2.9 ± 0.4 t ha^−1^ yr^−1^ under grazing and 4.3 ± 1.1 t ha^−1^ yr^−1^ under commercial agriculture in a relatively flat area of southern Zambia [71]. Higher rates (by about a factor of two) were recorded in a rugged area of Malawi [70], with particularly high rates under tobacco crops (approx. 22 t ha^−1^ yr^−1^). The differences highlight the important role of topography and other factors in soil erosion rates, alongside land use, and suggest that the main beneficiaries of this service are farmers in rugged areas with high erosion risk.

In extreme situations, erosion regulation can be crucial to the viability of cropping systems, as high rates of soil erosion have large impacts on crop yields. For example, removing the top 1 cm of soil (roughly 130 t ha^−1^) reduced yields by 14%, while removing the top 20 cm reduced yields by 75% in an erosion simulation experiment in semi-arid Zimbabwe [72]. It was notable that doubling the application of fertilizer was not able to compensate for the effect of even moderate erosion. Observations of real (rather than simulated) yield–erosion relationships are rare in the study region, but Hudson & Jackson (1959) showed a 27% reduction in maize yield in Zimbabwe as erosion increased from 0.3 to 6.0 t ha^−1^ (cited in [73]).

It is crucial to note that catchment scale studies show sediment yield rates (the mass of soil material leaving the catchment per hectare) roughly an order of magnitude lower than these plot scale erosion rates [71,74]. This implies that most eroded material is re-deposited within the catchment, creating winners and losers from erosion [75]. Despite this inter-catchment redistribution, more wooded catchments do show lower sediment yields [76], and Hecky *et al*. [74] suggest that agricultural catchments have sediment yields 10–33 times higher than the least disturbed catchments of Lake Malawi.

Soil erosion regulation also supports other ES; for instance, low sediment inputs to Lake Malawi help maintain its clear waters, which are crucial to many of the endemic fish species, some of which are important for the aquarium trade [70]. Likewise low sediment rates prolong the lifetime of dams providing hydroelectricity [77].

## 5. Hydrological services

In a region that experiences strong seasonal cycles in water availability, the role of woodlands in altering the timing, location and quality of water flows needs critical evaluation. For example, floods have displaced approximately 9 M people and caused $1.5 billion of damage over 29 years in the study region [78]. Meanwhile the highly seasonal water flows restrict the availability of water for human consumption and hydroelectricity generation [79].

Vegetation slows the passage of water through a catchment [80,81] and can enhance infiltration into the soil and ground water. However, the pan-tropical literature suggest there is little effect of woody vegetation on the functions that may be of use to people in the region: reducing flooding, enhancing groundwater recharge and enhancing dry season flows [80,82]. Under the intense and prolonged rainfall typical of tropical flooding events, any enhanced infiltration rates have minimal effects on flood generation [83]. The very sparse literature relating to the hydrology of African woodlands is inconclusive on the role of woodlands in supporting hydrological services. In one study, the modelled effects of converting woodland to agriculture were minimal [84], with most of the hydrological services being delivered by small areas of montane forest [85]. However, observations in the catchments adjacent to Lake Malawi provide some evidence of woodlands reducing the interannual variability in stream flow [74]. One aspect of woodland hydrology that has been relatively well studied is the role of dambos—shallow, seasonally inundated headwater wetlands common in southern Africa [86]. Studies show that different dambos can both increase and decrease dry season flows, flood responses and catchment evapotranspiration, with consensus only in the ability of dambos to moderate early wet season floods at a very small scale [86].

Modelling studies show that water recycled by vegetation is responsible for between 10 and 25% of precipitation in the region, except on the eastern coastal fringe—clearly an important service to the rain fed agriculture in the region [87]. The relative contribution to this recycling of trees, grasses and crops has not been studied, but trees (and thus woodlands) are likely to be important given their access to deeper soil moisture.

Overall, there are few studies that link the hydrological function of the dry tropics to impacts on human well-being, and this is particularly true in the miombo and mopane landscapes [88]. Research on this topic is needed, particularly in the context of altered tree water requirements and seasonal precipitation patterns under global change.

## 6. Global services

### (a) Carbon storage and cycling

The large area of southern African woodlands (table 3) means that they are important in global biogeochemical cycles, not least the carbon cycle. Current estimates of woody biomass based on global maps are unreliable in woodland regions [92,93], and more accurate methods of remotely sensing biomass have only been applied at a small scale to date [94]. Resorting to sparse field data, we estimate that the biomass stored in woodlands is between 18 and 24 PgC, split evenly between the soil and the woody vegetation (table 3). Thus, the woodlands store a comparable mass of carbon to the Congo basin rainforests (30 PgC; [95]), primarily due to their areal extent and higher soil stocks.

**Table 3.** Area, mean carbon area-density and carbon stocks in the woodlands of southern Africa. The area data come from different mapping and remote-sensing studies, while the carbon density data are from field studies (electronic supplementary material, table S3). The stocks are calculated as the product of area and density, with high and low variants based on the range of area estimates, and standard errors calculated based on the variability of the field studies.

	source		s.e.m.	notes
area		million km^2^		
	White (1983) [2]	3.05		total in southern Africa of miombo, mopane and undifferentiated woodland, based on pre-satellite era vegetation maps
	GLOBCOVER 2009 [89]	2.82		‘closed (more than 40%) broadleaved deciduous forest’ and ‘open (15–40%) broadleaved deciduous forest’ total in southern Africa
	GLC 2000 [90]	2.64		as above
	FRA 2010 [91]	2.25		‘forest’ area in the study area, based on national reports with different methods and classifications
carbon area-density		tC ha^−1^	tC ha^−1^	
	mean above-ground woody biomass	28.7	5.3	13 studies
	mean below-ground woody biomass	15.5	2.4	4 studies
	mean soil carbon stock (0–30 cm)	35.9	5.4	7 studies
	total	80.1	8.0	
soil and woody carbon stocks		PgC	PgC	
	high	24.4	2.4	
	low	18.0	1.8	

This store of carbon provides global benefits through its regulation of global climate change, providing the basis for several carbon offsetting projects in the region [96–99]. These projects are conceived as a way of allowing local people to be rewarded for the supply of these global services, but the degree to which they provide equitable benefits is contested and variable [100].

### (b) Other biodiversity related services

The high levels of biodiversity and endemism in the region [101,102] are not ES *per se* [103], but they underpin many of the services already discussed in this paper, as well as others of note. Some services are valuable even though they are yet to be realized, for example, diversity can increase the resilience of socio-ecological systems and offers a pool of mostly untapped genetic resources. Many ecosystems and species in the woodlands have high existence values, in that people derive well-being from the simple fact of knowing that they exist. These existence values are particularly high for charismatic mammals, and the central Zambezian miombo ecoregion has among the highest richness of terrestrial mammal species of all global ecoregions [104]. This includes species of very high conservation concern such as black rhinoceros as well as near-endemic large mammals like roan antelope and sable antelope. The opportunity to see such megafauna is one of the primary motivations for those visiting national parks in the region [105] and tourism is a major contributor to national economies in southern Africa (see below). Miombo and mopane woodlands also have a distinct avifauna which, although less diverse than southern African montane forest assemblages [106], includes a number of endemic species such as Miombo rock thrush, Shelley's sunbird and Lilian's lovebird, therefore attracting tourist revenue from birdwatchers.

## 7. Cultural services

Cultural ES [107] have generally received less attention than other ES, and the study region is no exception. At a local level, woodlands have substantial spiritual value in traditional African belief systems. Such belief systems are ‘profoundly ecological’ [108], and sacred groves have high cultural significance in many areas of southern Africa as grave sites and the dwelling places of powerful ancestral spirits [109]. Areas of woodland are used as grave sites in rural Malawi [110], while in Tanzania, sacred groves are used for initiating new community members and help to signify long-term land tenure [111]. Individual tree species may also have ritual importance: in many parts of Zimbabwe, *Parinari curatellifolia* is used to communicate with the ancestors and for the annual rainmaking ceremony [112], while *Pseudolachnostylis maprouneifolia* is considered sacred in southern Tanzania [113]. Medicinal plants, often classed as a provisioning service, also have a strong cultural component, particularly for health issues with no equivalent in ‘westernized’ medicine. In central Zimbabwe, for example, pegs of *Gardenia* spp. placed around the home prevent illnesses caused by witchcraft, while branches of *Peltophorum africanum* are used to shake water over the clothes of a deceased person to chase away evil spirits (R. Pritchard 2015, personal observation). Spiritual values and traditional ecological knowledge are known to vary widely within and between communities, contingent on (among other things) ethnicity, age and gender [114]; this heterogeneity in cultural values has not yet been adequately explored in southern Africa. Furthermore, changing religious beliefs and the declining influence of traditional leaders and spirit mediums may be diluting the perceived importance of sacred groves [109,111], and the impact of these changing belief systems on the cultural services derived from woodlands by local communities is not well understood.

Tourism is a major contributor to national economies in southern Africa, with international tourism receipts for the six countries covered by this paper totalling $3.7 billion in 2012 (http://data.worldbank.org). However, tourist revenue alone probably over-represents the cultural and aesthetic services visitors derive from woodlands. Of 60 National Parks in the region, only 14 and 13 have substantial areas of miombo or mopane, respectively. This may be because woodlands have a low carrying capacity for charismatic megafauna [115], meaning that, despite high mammalian species richness, the most valuable species are sparsely distributed and difficult to see in woodlands compared with open grasslands. Only regular visitors to wildlife areas express strong interest in bird and plant diversity, while large mammals are favoured by first time visitors [116]. Trophy hunting is a major contributor to tourist revenue in the region, and hunters in southern Africa have reported the emotional and spiritual connection to nature [117] and the opportunity to experience ‘wilderness' environments [118] as motivating factors for hunting. However, the concept of wilderness in Africa is complex and contested [119], and there is a paucity of studies examining landscape preferences and the aesthetic/existence values assigned to woodland landscapes by local communities, tourists and hunters.

## 8. The future of ecosystem services from southern African woodlands

A rigorous horizon scanning of the future of the ES described above would need to consider all of the potential indirect and direct drivers that might affect ecological processes, and human preferences and demand for ES [120], as well as changes to access and control over ES. To illustrate the complexity and diversity of these drivers, we summarize some that have been proposed in the literature (electronic supplementary material, table S4). Despite their abundance, complexity and interrelationships, the main drivers manifest themselves in a relatively small set of mechanisms that will impact woodland ES, namely: changed access to and control over ES (particularly provisioning services), various forms of land-use change (small holder agricultural expansion, small holder intensification, the expansion of large-scale commercial agriculture and peri-urban change), alterations to wood harvesting for energy, and environmentally determined changes to fundamental ecological processes (tree and grass growth, fire). This section summarizes what is known about these mechanisms of change and the impacts on the ES detailed above.

### (a) Environmental change and altered woodland ecology

Woodland ecology is governed by competition between trees and grasses for water and nutrients and frequent disturbance by fire, humans and in some areas, megaherbivores [5]. Climate change will likely lead to reduced plant available water during a shorter growing season, while the frequency of intense drought will increase (table 4). These conditions will likely lead to lower woody plant growth rates, and an increase in drought-driven mortality events that are currently rare. However, in opposition to predicted reductions in water availability, rising *p*CO_2_ will improve plant water use efficiencies [133], although to a lesser extent in C_4_ grasses and crops [134]. The net impact of these processes is unknown and is a priority for future research given model predictions suggesting an expansion of woodlands into grasslands [122].

**Table 4.** Future environmental changes and their likely impact on woodland ecological processes. RCPs, representative concentration pathways; LUC, land-use change; ENSO, El Niño-Southern Oscillation.

direct driver	future scenarios	likely impacts on woodlands	knowledge of impacts
rising concentration of atmospheric CO_2_ (*p*CO_2_)	by 2050 *p*CO_2_ increase to 443 ppm under low emissions (RCP2.6) by 2050 *p*CO_2_ increase to 541 ppm under low emissions (RCP8.5) [121]	increased water use efficiency particularly of trees; increased tree cover at grassland margins [122]; potentially altered species composition	not examined in miombo or mopane
rising temperatures^a^	0.5–1.9°C under low emissions (RCP2.6) 3.4–5.4°C under high emission scenario (RCP8.5) 90% CIs for southern Africa in 2100 [123]	reduced crop yields especially in high-yield systems [124] leads to LUC enhanced evaporation leads to less plant available moisture; altered woodland ecology, range shifts and species turnover, e.g. 30–47% range retraction for *Brachystegia spiciformis* in Zimbabwe and S. Mozambique [125]	well studied for crops, low for woodland ecology
changes in total annual precipitation^a^	−6% to +4% under low emissions −9% to +7% under high emissions; reductions in south and west of study region of up to 20% 90% CIs for southern Africa in 2100 [123]	under low emissions, impacts are likely to be minimal, but reduced rainfall under high emissions may lead to lower plant available moisture and productivity	precipitation manipulation experiments are needed
changes to seasonality of rainfall	lower rainfall at start of wet season (−15–37 mm mo^−1^), higher rainfall at end under RCP 8.5 [126]; predictions somewhat model dependent	shorter growing season for vegetation, increased soil erosion	well studied for crops, low for woodland ecology, especially phenological response [127]
change in ENSO	doubled frequency of intense El Niño over next century and associated droughts in southern areas of region [128,129]	unknown	drought response of woodland not studied
increased N deposition	increased NH*_x_* deposition under all RCPs [130]	enhanced plant growth rates in N limited areas	N addition response of woodland not studied
altered fire and other disturbance regimes	decline in burned area due to cropland expansion [131]	smaller fires may lead to less burned area and enhanced tree growth and recruitment [132]	several long-term fire experiments in the region provide valuable data; reviewed in [132]

^a^Data from Niang *et al.* [123], which are for all of the Southern African Development Community.

Predictions of future fire regimes show ambiguous results over the region [135,136]. In northern and western Africa, burned area has decreased as landscapes fragment due to agriculture. However in our study region, burned area variability appears to be driven by precipitation variation rather than land use [131]. Over the coming century, hotter, drier conditions may lead to more intense fires, but increasing tree growth over grasses could lead to a reduction in fuel. The role of disturbance by megaherbivores is already very limited [137] and will probably decline.

In summary, three scenarios of altered woodland ecology are plausible:

(i) Resistance but instability: climate change reduces the growing season, but this is matched by *p*CO_2_-driven increases in water use efficiency, albeit on mismatched time scales as projected reductions in plant water availability evolve over this century. The resultant woodland in 2100 may be similarly structured to today's, but plant species turnover and range shifts [125,138] are likely. These systems could be more variable through time due to more frequent or intense droughts [139,140].
(ii) Trees win: the direct effect of increasing *p*CO_2_ overrides or precedes the impact of climate change, generating increases in tree density, the transition of woodlands to forest and an expansion of trees into grass-dominated savannahs.
(iii) Productivity declines: the *p*CO_2_ effect may be weak compared with the climatic effects, because the potential for enhanced woody growth may be limited by other factors such as soil infertility and short growing seasons. Under this scenario, the whole system may become less productive, due to a shorter and shifted growing season and less available plant moisture, perhaps leading to phenological disruption and species turnover.

Several decades of remote-sensing data can potentially be used to assess whether any of these changes are underway. The results are divergent: two studies show increasing vegetation optical depth in some (different) parts of the region, which is interpreted as woody biomass increase [141,142]; studies based on the Normalized Difference Vegetation Index (from different sensors) show different results, with indications of no increases in most of the study area [143], and a reported increase in the amplitude of the annual NDVI cycle [144]. Three Leaf Area Index (LAI) products also show differing results, with one showing a decrease in growing season integrated LAI, and two showing statistically significant increases [145]. It is hard to infer anything other than data limitations from these studies, especially given the limited ground data to corroborate these observations [146].

This ecological uncertainty means that it is difficult to project the impacts on ES in the region: this appears to be beyond the scope of current scientific understanding, given the wide range of possible responses and feedbacks. A better understanding of woodland global change ecology is urgently needed.

### (b) Change in access to ecosystem services

Large changes to ES can occur without any change in ecosystem function if people's access to ecosystems changes [15]. One feature of the provisioning ES described above is that they are very widely used within rural communities suggesting that most rural people have *de facto* use rights to woodland, through state, communal or private property regimes [147]. Likely changes may be (i) the breakdown of traditional institutional arrangements leading to open access and (ii) the privatization of land currently under communal management (electronic supplementary material, table S4). Currently, little is known about how widespread different property regimes are, and their flux, but many case studies suggest that *de facto* access is commonly controlled by local, sometimes traditional, authorities [26,147]. Indeed it is only for high-value products that state control is common (timber and charcoal are the main examples [1]). Control is often affected through traditional institutions which can create mixtures of communal and private property regimes [148]. These regimes may for example restrict the timing and type of harvesting [149], prohibit felling of some tree species [26], or restrict charcoal production to the by-products of land clearance [148]. Such arrangements may break down because of a lack of outside recognition, the imposition of new arrangements by the state, changing attitudes to traditional religion [147], migration or other changes to rural communities' decision-making and power structures. This can lead to *de facto* open access, which can result in overuse and degradation of natural resources [150]. However, it should be cautioned that arrangements often appear to be open access when in fact more detailed study reveals elements of communal property regimes. Alongside the disruption of traditional institutional arrangements, there are several drivers that may lead to a replacement of communal property arrangements with private ones. These include most obviously the large-scale acquisition of land rights for commercial agriculture or conservation (see below). Another driver of privatization may be the increasing scarcity of woodland resources in some areas, which can lead to enclosure of fuelwood resources on private land, but the degree to which this occurs and for which ES appears very variable [151].

Large-scale afforestation is currently proposed for the region, associated for instance with conservation or climate change mitigation interests [12] and if this is realized, it would affect both the ecological provision of services and social access mechanisms. An example of this type of proposal is the World Resources Institute Atlas of Forest and Landscape Restoration Opportunities. Such initiatives are controversial in terms of their ecological premises [152], and depending on the nature of their implementation, stand to initiate significant changes in access to ES for local people's livelihoods [153]. Such access restrictions would compound the impacts of the expansion of large-scale agriculture (see below).

### (c) Wood harvesting for energy

Several drivers identified in the electronic supplementary material, table S4, suggest increased future demand for wood for energy [154]. This is linked to both population growth increasing aggregate demand, and urbanization leading to a switch from wood to charcoal (which requires more wood input for a given energy output), because longer woodfuel supply lines incentivize the use of energy dense charcoal [43,155]. On the other hand, consumers may switch to kerosene, gas or electricity or use more fuel-efficient stoves if price, accessibility and other barriers are addressed; this transition has been slow in the region to date and ‘fuel stacking’ means that a shift to cleaner-burning fuels rarely results in abandonment of previous energy sources and appliances [46,156]. On balance, total consumption of wood-based energy is likely to rise significantly: total energy use in the region rose at 2.8% yr^–1^ between 2000 and 2012 (IEA data, extrapolated to Malawi based on *per capita* usage for the other countries) while over the same period the proportion of this energy coming from woodfuels reduced only slightly from 81 to 76% (table 2). This increased demand for wood energy is unlikely to be met by imports or plantation forestry [157], so unless there are profound changes in the energy industry, woodlands are likely to be increasingly harvested for woodfuels.

Aggregate demand is not going to be a major driver of woodland conversion across the region in the near future, but hotspots of overharvesting are likely to persist and expand [19]. Current demand equates to 0.4 t ha^−1^ yr^−1^ (assuming the smallest area from table 3), which is well below reported regrowth rates of 1.1–2.2 t ha^−1^ yr^−1^ [115], but the heavy concentration of demand in the hotspots, where there are other demands for biomass, low woody resources and high demand can lead to fast moving and widespread degradation [155,158,159]. The 2.8% yr^−1^ growth in demand observed from 2000 to 2012 implies a quadrupling over 50 years, which would at that point likely exceed even the technical potential for regional renewable supply, given other uses for biomass and land-use change.

The main ES impacts of woodfuel harvesting will thus likely continue to be areas of high intensity harvesting near centres of demand, but if current trends continue, these supply areas will cover most of the region by the 2060s. Current regulation and licensing of woodfuels has been unable to prevent this frontier mode of exploitation [160], and this seems unlikely to change: political will to make large-scale changes to charcoal value chains is not apparent [41,161]. These woodfuel frontier areas are only slightly more distant from demand centres than those of market-orientated agriculture, so it seems likely that these areas will be under several pressures that may lead to land-cover change and altered ES. These issues need to be considered in the plans for transport and development corridors across the region.

Increased wood harvesting in these areas will alter ES provision in the woodlands, through its effect on ecosystem structure [162]. Harvesting leads to lower carbon storage, lower structural diversity, and can impact floral and faunal diversity [163,164]. The impact of harvesting is often transitory, with species richness and above-ground biomass returning to similar values after 20–30 years [164–166]. Critical to this pattern of succession is the post-harvest fire regime, land use and time to subsequent harvesting [167–170], with relatively few impacts on ES provision in situations where the harvest is highly selective [162] or where regrowth is not curtailed by alternate land uses [167].

### (d) Agricultural expansion and change

The main agricultural transitions in the region are: the expansion of small holder, often shifting, agriculture [171–174]; the intensification of small holder agriculture in areas of land scarcity [67], peri-urban areas and along transport corridors [175]; and the recent development of large-scale commercial cropping and animal husbandry, often linked to investments from abroad [176]. The importance of these processes varies across the region, with intensification taking place in Malawi, and probably other densely populated regions, large-scale land acquisitions most common in Tanzania and Mozambique (electronic supplementary material, figure S1), and the expansion of shifting cultivation presumably in areas of low population density (figure 1*a*).

Overall, these processes have led to modest gross deforestation across the region: 3.5% (0.06 M km^2^) of the 1.71 M km^2^ of woodland with more than 25% tree cover in 2000 was lost over 14 years [177]. This area of loss does not match in aggregate or per country with the increase in crop area reported in FAOSTAT of 0.10 M km^2^, half of which was in Tanzania (region-wide 2000 crop area = 0.170 M km^2^; 2014 = 0.270 M km^2^). The discrepancy probably reflects deficiencies in the crop area data, cropland expansion in non-forest areas, or classification and definitional issues with the woodland deforestation data. The regional data thus suggest modest crop area expansion and deforestation, in contradiction with several small-scale studies of land-cover change which have found much higher rates [94,174,178,179], but these are often located in hotspots of change. It should be noted that the reliability of the global Landsat-based deforestation data [177] has not yet been rigorously evaluated in woodlands—being notoriously hard to monitor due to intra- and interannual variations in land surface properties [180]. It also rests on a definition of forest/woodland that is not well connected to the ES provision of woodlands—it is very likely that much larger areas of woodland are undergoing change in biomass or species composition [159,169,174] without being defined as ‘forest loss’. Another change invisible to such analyses is agricultural intensification and a shift from subsistence to cash crops [63,181], a potential driver of change in woodlands because it can cause changes to the shifting cultivation system [182], and reduce the area under fallow. Fallow fields and woodland regeneration are an important source of landscape diversity and contain many species that are much used by humans [173].

The underlying drivers of these transitions, and the response and adaptation to them, differ greatly between countries, not surprising given their different political economy, history and current land cover and agrarian situation. For instance, poverty headcounts are falling in some countries but not all (data.worldbank.org/products/wdi); fertilizer use is rising fast in Tanzania and Malawi but is at 1960s levels in Zimbabwe and Mozambique (FAOSTAT); and *per capita* protein consumption increased by 50% in Angola and Mozambique, but remained constant in Tanzania, Zambia and Zimbabwe (FAOSTAT 1991–2000). The only drivers that appear to be consistent are the roughly linear increase in rural populations (region-wide from 25 M in 1950 to 103 M today), and the exponential increase in urban populations (1.4–52 M)—the region is predicted to be majority urban by 2048 [20]. Many processes will mediate the impact of these drivers on the woodlands, but, for example, the median of a large ensemble of land-use change projections suggest the cropland area in sub-Saharan Africa will increase by 50% by 2050 (however, the range spans no change and a doubling in crop area) [183].

Increasing connections to the global economy are evident in the rise in large-scale land acquisitions for commercial agriculture, although this is primarily occurring in Mozambique, and may have peaked in 2010 (electronic supplementary material, figure S1). These transactions have attracted controversy over both their social and environmental impacts; they currently occupy a small part of the landscape, but could account for a large proportion of future cropland expansion (intended and concluded land deals in the region cover 0.07 M km^2^ (2000–2015); electronic supplementary material, figure S5). A rapid switch to large-scale commercial agriculture is one common, but highly contested, vision of the route to development for the region. For example, the ‘miracle’ transformation of the Brazilian cerrado has been promoted as a development model for the Mozambican miombo (the ProSavanna project, see [184]). The notion that there are large areas of ‘unused’, ‘spare’ or ‘degraded’ land underpins this vision of commercial agricultural expansion [176], but these views are usually blind to the reality of shifting cultivation systems, or the role of grazing lands in supporting sedentary agriculture (see above). Thus, there are more barriers than might at first seem to be apparent to commercial expansion. However, the driving forces are growing, driven both endogenously through growing demand for meat and also via international linkages to new markets, often facilitated by aid, capital and technology transfer from more developed savannah regions [185].

## 9. Conclusion

Our synthesis builds on previous work [1,5] to show very strong linkages between ES provided by woodlands and the basic constituents of human well-being: *food* both directly and through the services that support crop and livestock raising; *fuel* for both urban and rural people; and construction materials. Only in relation to water regulation is the situation unclear—a crucial knowledge gap.

The provisioning services are relatively well studied and synthesized [26,57,186], and recent livelihood analyses have made their importance to the rural poor clear (table 2). By contrast, the regulating and supporting services are not well studied; for instance, it is only in Zimbabwe that the nutrient flows from woodland to farm have been well quantified and in general the link to shifting cultivation is often neglected, not least because the number of shifting cultivators is unknown. The carbon storage of the woodlands has been examined, but large uncertainties remain in estimating fluxes and rates of change.

Key processes that are currently changing ES in the region (table 4) are the expansion of smallholder and large-scale agriculture, the harvesting of biomass for wood energy, and altered access to and management of ES. Climate change and rising CO_2_ may alter woodland ecology in the future and impact ES, but current understanding provides little constraint on future ecology—leaving open the potential for surprises. The land-use change processes are likely to be the major force in altered ES in the near future [187], and there is a particular need to understand how land-use and land-cover changes will alter the ES that support the livelihoods of the rural poor, and how they will adapt.

Current rates of crop expansion and deforestation appear to be modest, but the existing drivers of land-use change are accelerating, whilst new drivers and processes are becoming more important as the woodlands are connected to the global land, financial and energy systems. Several crucial land-cover and land-use transitions are poorly quantified, including agricultural intensification, the decline of shifting cultivation and floristic and structural changes to the woodlands. Furthermore, there is virtually no systematic data on the tenure arrangements that currently allow a broad section of society to access woodland ES, and no region-wide view on how these arrangements are changing. Ecological knowledge about ecosystem function is virtually meaningless to the poverty alleviation debate unless we understand *who* has the ability and rights to use ES, and how this is changing [17].

## Supplementary Material

Supplementary Tables and Figures
