# Supplementary material for: Ecosystem services from southern African woodlands and their future under global change
Source: Philos Trans R Soc Lond B Biol Sci. 2016 Sep 19;371(1703):20150312. doi: 10.1098/rstb.2015.0312 (PMC4978870; doi:10.1098/rstb.2015.0312)
Supplement: Supplementary Tables and Figures [file rstb20150312supp1.docx]

# Supplementary tables and figures

## Contents

Table S1: A summary of the main provisioning services from woodlands

Table S2: Absolute and relative income derived from wild land resources in the miombo and mopane region

Table S3: Indirect and direct drivers of change in ecosystem service provision in the miombo and mopane region, and the mechanisms by which they may alter service provision

Table S4. Vegetation (a) and soil (b) carbon stocks in miombo and mopane woodlands

Figure S1: Trends and distribution of land acquisitions in the miombo and mopane region

Table S1: A summary of the importance of the main provisioning services from woodlands, with a ranking of their importance to a range of beneficiaries. More detailed records of wild species collected as food can be found in Malaisse (2010) and McGregor (1995), working in Tanzania and Zimbabwe respectively.

| **Product** | **Beneficiary** | | | | | **Notes** |
| --- | --- | --- | --- | --- | --- | --- |
|  | **Local use as a safety net** | **Local subsistence consumption** | **Rural markets** | **Urban/regional markets** | **International** |  |
| **Wild Foods** | | | | | | |
| *Wild Fruits* | High | High | Medium | Medium | Medium | Over 50 fruit-bearing trees are commonly used by rural households in miombo regions (Maghembe et al., 1994). Most are for subsistence consumption. *Parinari curatellifolia* is particularly important during droughts. Species including *Vitex payos, Uapaca kirkiana* and *Mimusops zeyheri* have been recorded sold locally and regionally (Rossiter et al., 1997; Karaan et al., 2005). Only *Adansonia digitata* and *Sclerocarya birrea* are used to create products widely sold internationally. Naturalised species such as *Psidium guajava* and *Ziziphus mauritania* are also widely used. |
| *Wild Vegetables* | Medium | Medium | Low | No reports | No reports | Malaisse (1994) recorded 50 species of edible leaves in Tanzania, and McGregor (1995) recorded 39 wild vegetable species in Zimbabwe, of which the most regularly consumed were *Cleome gynandra* and *Corchorus tridens*. Local sales of dried wild vegetables have been observed in Zimbabwe. |
| *Mushrooms* | Low | Medium | Medium | Low | No reports | Edible mushrooms are most often from the genera *Cantharellus, Termitomyces, Amanita* and *Russula*.45 edible species were recorded in Zimbabwe (Wilson, 1990, in McGregor 1995) and 60 in Malawi (Makonda & Gillah, 2007).  Local and regional mushroom sales have been recorded in Zimbabwe, Zambia (Pegler & Piearce, 1980) and Malawi (Lowore, 2006). However, the short growing season means mushrooms can make only a small contribution to rural livelihoods. |
| *Edible insects* | Medium | Medium | Medium | Medium | Low | Edible insects, including termites, grasshoppers, locusts and caterpillars, are widely consumed as snacks and relishes in southern Africa, but their economic and nutritional importance to rural households has not been well quantified.  Mopane worms (*Gonimbrasia belina*) are sold in Zambia (Silow, 1976; Mbata, 1995), Malawi ( Munthali & Mughogho, 1992) and Zimbabwe (Stack et al., 2003). The caterpillars *Gynanisa maja* and *Gonimbrasia zambesina* are also sold locally and regionally in Zambia (Mbata et al. (2002). |
| *Honey* | Low | Low | Medium | Medium | Low but increasing | Honey from the miombo ecoregion is sold locally and regionally, with increasing levels of international export particularly in Mozambique and Zambia (Campbell et al., 1997; Mickels-Kokwe, 2006; Timko, 2013). However at present honey production is quite localised, with lack of market access (Wiersum & Endamalaw, 2013) and poor recognition of honey producers’ woodland access rights (Wily & Mbaya, 2001; Hausser & Mpuya, 2004) limiting expansion in production. |
| *Bush meat* | Medium | High | Medium | Medium | Low | The importance of bush meat to rural households in the miombo region is poorly understood. The existing literature (reviewed by Lindsey et al., 2013) focuses on the ecological impacts of illegal large game hunting in national parks. Such game is often sold locally or regionally rather than consumed in the home (Carpanetto & Fusari, 2000) and hunters have been found to earn more than non-hunters in Tanzania (Knapp, 2007) and Zambia (Brown, 2007). Much less well documented is the capture of small game by rural households, such as mice, birds and rabbits. |
| **Building and Craft Materials** | | | | | | |
| *Barks and Fibres* | Low | Medium | Medium | Medium | No reports | The bark of *Adansonia digitata* is used to make crafts (Luckert et al., 2001) and households in the Save Odzi Valley in Zimbabwe sell crafts as part of drought coping strategies (Romero et al., 2001). The fibres of indigenous trees such as *Brachystegia boehmii* and *B.spiciformis* are sometimes used for thatching houses and (more rarely) for rope making. |
| *Thatching grass* | Medium | High | High | Medium | No reports | Grasses (most often of genus *Hyparrhenia*) are used for thatching household structures. Thatching grasses are normally collected by women (Kalaba et al., 2013, although see Timko, 2013) and in central Zimbabwe thatching grass sales were described as an important method for coping with food shortages and raising money for school fees. |
| *Construction Poles** | Low | High | Medium | Low | No reports | Wooden poles are used in numerous structures in rural homesteads including houses, granaries, livestock pens and fences. The majority of poles are collected for use by the household (Grundy et al., 1993), although some trading of poles has been recorded in both informal and urban markets (Attwell et al., 1988; Luoga et al., 2000) |
| **Medicinal Plants** | | | | | | |
| *Medicinal plants* | Low | High | High | High | Medium | Medicinal plants are crucially important to rural households in the miombo region. Gelfand et al. (1985) estimate that 10% of the over 5000 native plants in Zimbabwe have medicinal uses, while Medimoa (1983) described 585 plants with medicinal uses in Mozambique. Medicinal plants are traded locally, regionally and internationally (Botha et al., 2004). |

*Construction poles refers to small-scale collection, and does not include industrial-scale commercial timber harvesting.

## References for Table S1

Attwell, C.A.M, Campbell, B.M., du Toit, R.F. & Lynham, T.J.P. (1988) Patterns of fuelwood utilisation in Harare, Zimbabwe. Harare: Forestry Commission of Zimbabwe

Botha, J., Witkoswki, E.T.F. & Shackleton, C.M. (2004) Market profiles and trade in medicinal plants in the Lowveld, South Africa. *Environmental Conservation* **31**: 38-46

Brown, D. (2007) Is the best the enemy of the good? Institutional and livelihoods perspectives on bushmeat harvesting and trade: some issues and challenges. In: *Anonymous Bushmeat and Livelihoods: Wildlife Management and Poverty Reduction*, eds Davies, G. & Brown, D. Oxford: Blackwell Publishing Ltd

Carpaneto, G.M. & Fusari, A. (2000) Subsistence hunting and bushmeat exploitation in central-western Tanzania. *Biodiversity and Conservation* **9**: 1571-1585

Gelfand, M. (1985) The traditional medical practitioner in Zimbabwe: his principles of practice and pharmacopoeia. Gweru: Mambo Press

Grundy, I.M., Campbell, B.M., Balebereho, S. et al. (1993) Availability and use of trees in Mutanda Resettlement Area, Zimbabwe. *Forest Ecology and Management* **56**: 243-266

Hausser, Y. & Mpuya, P. (2004) Beekeeping in Tanzania: When the bees get out of the woods – an innovative cross sectoral approach to community based natural resource management. *Game and Wildlife Science* **21**: 291-312

Kalaba, F.K., Quinn, C.H. & Dougill, A.J. (2013) Contribution of forest provisioning ecosystem services to rural livelihoods in the Miombo woodlands of Zambia. *Population and Environment* **35**: 159-182

Karaan, M., Ham, C., Akinnifesi, F., Moombe, K., Kordaan, D., Franzel, S. & Aithal, A. (2005) Baseline marketing surveys and supply chain studies for indigenous fruit markets in Tanzania, Zimbabwe and Zambia. World Agroforestry and CPWild Research Alliance.

Knapp, E.J. (2007) Who poaches? Household economies of illegal hunters in Western Serengeti, Tanzania. *Human Dimensions of Wildlife* **12**: 195-196

Lindsey, P.A., Balme, G., Becker, M. et al. (2013) The bushmeat trade in African savannas: impacts, drivers and possible solutions. *Biological Conservation* **160**: 80-96

Lowore, J. (2006) Miombo woodlands and rural livelihoods in Malawi. CIFOR: Bogor, Indonesia

Luckert, M.K., Nemarundwe, L., Gibbs, L., Grundy, I., Hauer, D., Maruzane, D., Shackleton, S. & Sithole, J. (2001) Contribution of baobab production activities to household livelihoods. In: Household livelihoods, marketing and resource impacts: A case study of bark products in Eastern Zimbabwe. IES Working Paper 18, University of Zimbabwe, Harare.

Luoga, E.J., Witkowski, E.T.F. & Balkwill, K. (2000a) Subsistence use of wood products and shifting cultivation within a miombo woodland of eastern Tanzania, with some notes on commercial uses. *South Africa Journal of Botany* **66**: 72-85

Maghembe, J.A., Kwesiga, F., Ngulube, M., Prins, H. & Malaya, F.M. (1994) Domestication potential of indigenous fruit trees of the miombo woodlands of southern Africa. In: *Tropical trees: the potential domestication and the rebuilding of forest resources*. Eds. Leakey, R.R.B. & Newton, A.C. Institute of Terrestrial Ecology, Natural Environment Research Council, UK.

Makonda, F.B.S. & Gillah, P.R. (2007) Balancing wood and non-wood products in miombo woodlands. Working Papers of the Finnish Forest Research Institute 50. Available at <http://www.metla.fi/julkaisut/workingpapers/2007/mwp050-07.pdf>. Last accessed 21.4.16

Malaisse, F. (2010) How to live and survive in Zambezian Open Forest (Miombo Ecoregion). Gembloux: Les Presses Agronomiques de Gembloux

Mbata, K.J. (1995) Traditional uses of arthropods in Zambia: I: The food insects. *The Food Insects Newsletter* **8**: 5-7 – no pdf

Mbata., K.J., Chidumayo, E.N. & Lwatula, C.M. (2002) Traditional regulation of edible caterpillar exploitation in the Kopa area of Mpika district in northern Zambia. *Journal of Insect Conservation* **6**: 115-130

McGregor, J. (1995) Gathered produce in Zimbabwe’s communal areas changing resource availability and use. *Ecology of Food and Nutrition* **33**: 163-193

Medimoa, E.E. (1983) Medicinal plants from Mozambique Minerva. Maputo: Central Press

Mickels-Kokwe, G. (2006) Small-scale woodland-based enterprises with outstanding economic potential: the case of honey in Zambia. Centre for International Forestry Research, Bogor, Indonesia

Munthali, F.X. & Mughogho, D.E.C. (1992) Economic incentives for conservation: bee-keeping and Saturnidae caterpillar utilisation by rural communities. *Biodiversity and Conservation* **1**: 143-154

Pegler, D.N. & Piearce, G.D. (1980) The edible mushrooms of Zambia*. Kew Bulletin* **35**: 475-491

Romero, C., Dovie, D., Gambiza, J., Luoga, E., Schmitt, S. & Grundy, I. (2001) Effects of commercial bark harvesting on *Adansonia digitata* (Baobab) in the Save Odzi-valley, Zimbabwe with considerations for its management. In: Household livelihoods, marketing and resource impacts: A case study of bark products in Eastern Zimbabwe. IES Working Paper 18, University of Zimbabwe, Harare.

Rossiter, S., Pellegrin, S., Hill, K., Bunting, A., Robinson, S., Mason, T., Boitumelo, T. & Madiba, A. (1997) The present importance and future potential of veld products in the livelihoods of villagers from the Tswapong Hills. Botswana Notes and Records *29*: 9-22

Silow, C.A. (1976) Edible and other insects of mid-western Zambia. Studies in ethno-entomology. Uppsala: Institutionen för allmän och Jämförande Ethnografi vid Uppsala Universitet

Stack, J., Dorward, A., Gondo, T., Frost, P., Taylor, F. & Kurebgaseka., N. (2003) Mopane worm utilisation and rural livelihoods in Southern Africa. Paper presented at the International Conference on Rural Livelihoods, Forests and Biodiversity, 19-23 May 2003, Bonn, Germany

Timko, A. (2013) Exploring forest-related coping strategies for alleviating the HIV/AIDS burden on rural Malawian households. *International Forestry Review* **15**: 230-240

Wiersum, K.F. & Endamalaw, T.B. (2013) Governing forests for provisioning services: the example of honey production in southwest Ethiopia. In: Governing the Provision of Ecosystem Services, eds Muaradian, R. & Rival, L. *Studies in Ecological Economics Volume 4.* Dordrecht: Springer

Wily, L.A. & Mbaya, S. (2001) Land, people and forest in eastern and southern Africa at the beginning of the 21^st^ century. The impact of land relations on the role of communities in forest future. IUCN Eastern Africa Programme, Forests and Social Perspectives in Conservation, NRI: IUCN, Nairobi

Table S2. Absolute income and relative income dependency (percentage of household income) derived from wild land resources in the miombo and mopane region. Income is separated into wood fuels and non-timber woodland products, with the remainder largely comprising construction materials such as wooden poles. Income includes both cash income and imputed prices for products consumed within the household. Average values are scaled to the regional totals using the mean hh size in the studies (5.9±0.2 people per hh). PP$ are US dollars adjusted to 2016 values and for purchasing power parity in the relevant countries. Updated from Shackleton & Gumbo (2010).

| **Country** | **Year(s) Data Collected** | **Woodland Type** | **Location** | **Income from wild lands (cash and consumption, PPP$US household-1 yr-1)** | | | **Wild land income as % of total income (cash and consumption)** | | | **Ref** | **Notes** |
| --- | --- | --- | --- | --- | --- | --- | --- | --- | --- | --- | --- |
|  |  |  |  | **Total** | **of which woodfuel** | **of which NWFPs** | **Total** | **of which woodfuel** | **of which NWFPs** |  |  |
| Zimbabwe | 1993-1994 | Miombo/ Mopane | Shindi Ward, Chivi District | 348 | 90 | 258 | 28 | 7 | 21 | Cavendish 1999; 2000 | Defines environmental income as ‘all resources freely provided by natural processes.’ Environmental income figure reported here excludes gold, but includes other soils e.g. building and painting materials. Woodfuel figures are only for household consumption – although some households may have also sold firewood, this is unlikely to have been a major contributor to livelihoods in the study region and so will have had limited impact on the accuracy of the figures. |
|  | 1996-1997 |  |  | 471 | 114 | 357 | 34 | 8 | 25 |  |  |
| Zimbabwe | 1993 | Arid-eutrophic savanna | Jinga | 800 |  |  |  |  |  | Campbell, Luckert and Scoones 1997 | The only study to deduct labour costs from environmental resource income – per household income was adjusted using the authors’ own estimate that labour costs account for approximately 75% of total product value, but this may have resulted in overestimation of environmental income. |
|  |  | Miombo | Matendeuze | 486 |  |  |  |  |  |  |  |
| Zimbabwe | 1999-2000 | Miombo | Romwe, Chivi District | 357 | 124 | 132 | 12 | 4 | 4 | Campbell, Jeffrey, Kozanayi, Luckert, Mutamba & Zindi 2002 | The only study reporting total gross income, rather than net income. May have resulted in underestimation of the relative contribution of environmental income compared to other studies as crop and livestock production have high input costs compared to environmental resources. |
|  |  | Mopane | Mutangi, Chivi District | 399 | 155 | 149 | 14 | 5 | 5 |  |  |
| Malawi | 2006-2007 | Miombo, montane | Liwonde Forest Reserve | 98 |  |  | 16 |  |  | Chilongo 2014 | Part of the CIFOR-PEN global study (Angelsen et al., 2014) and used PEN technical guidelines (PEN, 2007). |
|  |  | Miombo, montane | Chimaliro Forest Reserve | 86 |  |  | 10 |  |  |  |  |
| Tanzania | 2010 | Miombo | Kilosa District | 190 | 80 | 22 | 19 | 9 | 2 | Dokken & Angelsen 2015 | Not a CIFOR-PEN study, but used PEN methodologies. |
| Malawi | 1999-2000 | Miombo, montane | Mulanje Mountain Reserve | 406 |  |  | 33 |  |  | Fisher 2004 | Environmental income defined as forest related cash earnings plus value of home-consumed firewood, so may be an underestimate due to exclusion of other home-consumed environmental products. |
| Mozambique | 2006 | Miombo | Gorongosa NP buffer zone, Sofala | 802 | 219 | 341 | 37 | 10 | 16 | Hegde & Bull 2008 | Part of the CIFOR-PEN global study (Angelsen et al., 2014) and used PEN technical guidelines (PEN, 2007). |
| Zambia | 2005 | Miombo | Paul Kalemba | 418 |  |  | 26 |  |  | Jumbe, Bwalya & Husselman 2007, mean of 7 sites |  |
| Malawi | 2004 | Miombo, montane and eucalyptus | Chiradzulu District | 192 | 144 | 32 | 12 | 9 | 2 | Kamanga, Vedeld & Sjaastad 2009 | Includes all non-cultivated products from common property forest (figures are also given for resources obtained from privately owned forest, but these are not included here). |
| Zambia | 2005 | Miombo | Mufulira, Copperbelt | 1958 |  |  | 50 |  |  | Mutamba 2007 | No clear definition given of forest income. Although data are not disaggregated by product type, high contribution of forest resources to livelihoods likely to be because both study sites have good access to markets and a thriving charcoal trade. |
|  |  | Miombo | Kabompo, Northwestern | 1038 |  |  | 51 |  |  |  |  |
| Tanzania | 2007 | Miombo | Urumwa Forest Reserve | 904 | 512 | 160 | 46 | 26 | 8 | Njana, Kajemba & Malimbwi 2013 | No clear definition given of forest income. Charcoal production and tobacco farming are important industries in the area, explaining the relative importance of firewood in rural livelihoods. |
| Tanzania | 2007 | Miombo and montane | Mikumi National Park | 182 | 179 | 3 | 6 | 6 | 0 | Vedeld, Juamne, Wapalila & Songorwa 2012 | Study carried out close to a protected area – low contribution of environmental resources may have been due to strict access restrictions. |
| Mozambique | 2006-2007 | Miombo | Munhinga & Pindanganga, Manica | 145 |  |  | 24 |  |  | Walelign & Oystein 2013 | Part of the CIFOR-PEN global study (Angelsen et al., 2014) and used PEN technical guidelines (PEN, 2007). |
|  |  |  |  |  |  |  |  |  |  |  |  |
|  |  |  | Regional mean (PPP$) | 516 ±108 | 180 ±44 | 161 ±44 | 26 ±4% | 9 ±2% | 9 ±3% |  |  |
|  |  |  | Regional total (Billion $) | 8.74 ±1.83 | 3.04 ±0.75 | 2.74 ±0.75 |  |  |  |  |  |

Table S2 builds on the work of Shackleton and Gumbo (2010; Table 4.1 , pp. 70) with a detailed literature review, including all studies found to fulfil the following criteria: (a) the study site is in one of the six miombo eco-region countries which are the focus of this paper; (b) income includes both cash income and imputed values of products (both environmental and agricultural) consumed within the household; (c) contribution of wild land resources to household economies can be deduced both in absolute value terms and (in all but one case) as a percentage contribution to total household annual income. Following Dokken and Angelsen (2015), figures were converted to PPP$US using conversion factors from <http://www.econstats.com/weo/V013.htm> and expressed per household. Where studies did not provide data on mean household size, the mean household size of 5.9±0.2 people per household across all the studies was used to convert per capita data.

The table should be interpreted with a number of caveats in mind. Firstly, the definitions of environmental income and NTFPs are not consistent across all studies. Where possible mineral resources such as gold and soils (for painting and flooring) have been excluded from environmental income figures, but there was not always sufficient data disaggregation in the published papers for this to be possible. Secondly, there is variation in income accounting techniques and environmental resource valuation methodologies (the problems caused by lack of standardisation in environmental income accounting methods are well reviewed by Vedeld et al., 2004). Thirdly, local contextual factors (such as market availability and proximity to protected areas) can have a strong impact both on environmental income and on the accuracy of data collection. The potential impact of these methodological considerations on the validity of each environmental income estimate is detailed in the table.

## References for table S2

Angelsen, A., Jagger, P., Babigumira, R. et al. (2014) Environmental income and rural livelihoods: a global-comparative analysis. World Development 64: S12-S28

Campbell, B.M., Luckert, M. & Scoones, I. (1997) Local-level valuation of savanna resources: a case study from Zimbabwe. Economic Botany 51: 59-77

Campbell, B.M., Jeffrey, S., Kozanayi, W. et al. (2002) Household livelihoods in semi-arid regions: options and constraints. Centre for International Forestry Research, Bogor, Indonesia.

Cavendish (2000) Empirical regularities in the poverty-environment relationship of rural households: evidence from Zimbabwe. World Development 28: 1979-2003

Cavendish (1999) Incomes and poverty in rural Zimbabwe during adjustment: the case of Shindi Ward, Chivi Communal Area, 1993/94 to 1996/97. DfiD Southern Africa, Oxford, UK

Chilongo (2014) Livelihood strategies and forest reliance in Malawi. Forests, Trees and Livelihoods 23: 188-210 – PEN study

Dokken, T. & Angelsen, A. (2015) Forest reliance across poverty groups in Tanzania. Ecological Economics 117: 203-211 – PEN definitions, although not timings

Fisher, M. (2004) Household welfare and forest dependence in southern Malawi. Environment and Development Economics 9: 135-154

Hegde, R. & Bull, G. (2008) Economic shocks and miombo woodland resource use: A household level study in Mozambique. In: Managing the Miombo woodlands of Southern Africa: policies, incentives and options for the rural poor. The World Bank, Washington DC, USA (2008) 80-105 – PEN definitions

Jumbe, C.B.L., Bwalya, S.M. & Husselman, M. (2007) Contribution of dry forests to rural livelihoods and the national economy in Zambia. Available at <http://www.cifor.org/miombo/docs/ZambiaNationalCaseStudy.pdf> , last accessed 25.4.16

Kamanga, P., Vedeld, P. & Sjaastad, E. (2009) Forest incomes and rural livelihoods in Chiradzulu District, Malawi. Ecological Economics 68: 613-624

Mutamba, M. (2007) Farming or foraging? Rural livelihoods in Mafulira and Kabompo districts of Zambia. Paper presented at the Workshop Policies and Incentives of Miombo Woodland Management, October 30-31, Lusaka, Zambia.

Njana, M.A., Kajembe, G.C. & Malimbwi, R.E. (2013) Are miombo woodlands vital to the livelihoods of rural households? Evidence from Urumwa and surrounding communities, Tabora, Tanzania. Forests, Trees and Livelihoods 22: 124-140

PEN (2007) PEN technical guidelines version 4, Poverty Environment Network, CIFOR, Bogor, Indonesia.

Vedeld, P., Angelsen, A., Sjaasatd, E. & Berg, G.K. (2004) Counting on the environment: forest incomes and the rural poor. World Bank, Washington, D.C.

Vedeld, P. & Abdallah, J.M. (2012) Protected areas, poverty and conflicts: a livelihood case study of Mikumi National Park, Tanzania. Forest Policy and Economics 21: 20-31

Walelign, S.Z. & Oystein, J.N. (2013) Seasonal household income dependency on forest and environmental resources in rural Mozambique. International Journal of AgriScience 3: 91-99 – PEN study

Tables S3. Vegetation (a) and soil (b) carbon stocks in Miombo and Mopane woodlands. One value for belowground woody biomass was excluded from the mean as it seemed implausible (marked with an asterisk). NB: This does not represent a good sample of the woodland region, but does cover the main countries, with the exception of Angola. The studies are biased towards locations in forest reserves and other protected areas.

| Source | Location | Woodland type | AGB ± standard error of the mean (tC/ha) | BGB ± standard error of the mean (tC/ha) | Notes |
| --- | --- | --- | --- | --- | --- |
| (Ribeiro et al. 2008) | Mozambique | miombo | 28.4 ± 2.55 |  | Niassa National park |
| (Ryan et al. 2011) | Mozambique | various | 21.2 ± 1.4 | 8.5±0.5 | Includes degraded woodland |
| (Kutsch et al. 2011) | Zambia | miombo | 59.6±41.8 |  | 1 Degraded plot and 3 in forest reserve |
| (Shirima et al. 2011) | Tanzania | miombo | 23.3±9.8 |  | Nyanganje Forest Reserve |
| (Malimbwi et al. 1994) | Tanzania | miombo | 13.2 | 3.3* | degraded; Kitulangalo forest reserve |
| (Frost & Campbell 1996) | Zaire | miombo | 72 | 17.5 |  |
|  | Central Zambia | miombo | 34.9 | 16.4 |  |
| (Chidumayo 1997) | Zambia | miombo | 33.6 | 19.4 |  |
| (Guy 1981) | Zimbabwe | miombo | 5.9 |  | Sengwa Wildlife Research Area |
|  |  | Riverine woodland | 13.4 |  |  |
|  |  | Riverine woodland | 9.6 |  |  |
|  |  | Riverine woodland | 26.6 |  |  |
|  |  | Mopane | 30.9 |  |  |
|  |  |  |  |  |  |
| **Mean** |  |  | **28.7** | **15.5** |  |
| Sample size |  |  | 13 | 4 |  |
| Standard error of the mean |  |  | 5.3 | 2.4 |  |

| Source | Country | Soil Carbon Stocks tC/ha (0-30 cm) |
| --- | --- | --- |
| (Walker & Desanker 2004) | Malawi | 49.1 |
| (King & Campbell 1994) | Zimbabwe | 29.9 |
| (Williams et al. 2008) | Mozambique | 57.9 |
| (Woollen et al. 2012) | Mozambique | 40.1 |
| (Winowiecki et al. 2016) | Tanzania | 33.3 |
| (Cambule et al. 2014) | Mozambique – mopane/combretum | 24.6 |
| (Cambule et al. 2014) | Mozambique – mopane shrubveld | 16.2 |
|  |  |  |
| **Mean** |  | 35.9 |
| Sample size |  | 7 |
| Standard error of the mean |  | 5.4 |

## References for tables S3a and S3b

Chidumayo, E.N., 1997. Miombo ecology and management : an introduction, London: IT Publications in association with the Stockholm Environment Institute.

Cambule AH, Rossiter DG, Stoorvogel JJ, Smaling EMA (2014) Soil organic carbon stocks in the limpopo national park, mozambique: Amount, spatial distribution and uncertainty. Geoderma, 213, 46–56.

Frost, P. & Campbell, B.M., 1996. The ecology of Miombo woodlands. In The Miombo in transition: Woodlands and welfare in Africa. Bogor, Indonesia: Center for International Forestry Research, pp. 11–55.

Guy, P.R.P.R., 1981. Changes in the Biomass and Productivity of Woodlands in the Sengwa Wildlife Research Area, Zimbabwe. Journal of Applied Ecology, 18(2), pp.507–519.

King, J.A. & Campbell, B.M., 1994. Soil organic matter relations in five land cover types in the miombo region (Zimbabwe). Forest Ecology and Management, 67(1-3), pp.225–239.

Kutsch, W.L. et al., 2011. The Charcoal Trap: Miombo Forests and the Energy Needs of People. Carbon balance and management, 6(1), p.5.

Malimbwi, R.E., Solberg, B. & Luoga, E., 1994. Estimation of biomass and volume in miombo woodland at Kitulangalo Forest Reserve, Tanzania. Journal of Tropical Forest Science, 7(2), pp.230–242.

Ribeiro, N.S.N. et al., 2008. Aboveground biomass and leaf area index (LAI) mapping for Niassa Reserve, northern Mozambique. Journal of Geophysical Research, 113(G3), pp.1–12.

Ryan, C.M., Williams, M. & Grace, J., 2011. Above-and Belowground Carbon Stocks in a Miombo Woodland Landscape of Mozambique. Biotropica, pp.1–10.

Shirima, D.D. et al., 2011. Carbon storage, structure and composition of miombo woodlands in Tanzania’s Eastern Arc Mountains. African Journal of Ecology, 49(3), pp.332–342.

Walker, S.M. & Desanker, P. V, 2004. The impact of land use on soil carbon in Miombo Woodlands of Malawi. Forest Ecology And Management, 203(1-3), pp.345–360.

Williams, M. et al., 2008. Carbon sequestration and biodiversity of re-growing miombo woodlands in Mozambique. Forest Ecology And Management, 254(2), pp.145–155.

Winowiecki L, Vagen T-G, Huising J (2016) Effects of land cover on ecosystem services in Tanzania: A spatial assessment of soil organic carbon. *Geoderma*, **263**, 274–283.

Woollen, E., Ryan, C.M. & Williams, M., 2012. Carbon Stocks in an African Woodland Landscape: Spatial Distributions and Scales of Variation. Ecosystems.

Table S4. Indirect and direct drivers of change in ecosystem service provision in the miombo and mopane region, and the mechanisms by which they may alter service provision. For clarity the interacting and multiple links between underlying drivers and direct drivers are not shown, nor the feedbacks between drivers or the feedbacks from the mechanisms of change to the drivers. The table is not designed to be comprehensive, but illustrates many of the processes discussed in the literature. Their importance will be variable in time and space.

| **Indirect global change drivers** | **Pressures / direct drivers** | **Ref** | **Mechanism of influence on ES** |
| --- | --- | --- | --- |
| Demographic change | Rising demand for staple foods | (UNPD 2014) | LUC |
|  | Rising demand for energy | (UNPD 2014) | Wood harvesting |
|  | Rising rural pop leads to induced intensification and a decline in shifting cultivation | (Pingali et al. 1988; van Vliet et al. 2012; McNicol et al. 2015) | LUC, biodiversity change |
| Poverty, Economic Development & Urbanisation | Urbanisation without industrialisation means a lack of non-farm well-paid jobs and continued dependence on peri-urban land for fuel and food. | (Rudel 2013) | Peri-urban and transport corridor LUC |
|  | Urbanisation reduces rural population growth and the expansion of small scale agriculture | (UNPD 2014) | Remote rural LUC; peri-Urban LUC |
|  | Urbanisation leads to increased demand for charcoal over wood (longer transport distances) | (Mwampamba 2007; Zulu 2010; Sander et al. 2013) | Wood harvesting |
|  | Urban electricity and gas supply may improve and fall in price |  |  |
|  | As income rises, so does expenditure on meat and fat | (Alcamo et al. 2005) | Agricultural change to meat production or feedcrops, biodiversity change from bushmeat |
|  | Physical access to the remote woodlands improves with creation of new transport corridors | (Laurance et al. 2014) | Wood harvesting, LUC |
|  | Market access improves for both agricultural inputs and products leading to agric diversification and intensification | ??? | Agricultural change and other LUC |
|  | Increased extractive industries causes “Dutch Disease” and makes agriculture less profitable via labour costs and currency effects | (Rudel 2013) | LUC |
| Technological Change | New agricultural technology makes commercial agriculture viable on poor soils and increases cattle ranching in tsetse prone areas | (Gasparri et al. 2016) | LUC |
|  | Increased efficiencies in production and consumption of wood fuels | (Owen et al. 2013) | Wood harvesting |
| Social, Cultural, and Political Drivers | Globalisation leads to increased connections to new commodity markets e.g. beef, soya, sesame, biofuels | (Anseeuw et al. 2013; Messerli et al. 2014) | LUC |
|  | Better connections to global markets increases imports of food and energy, reducing demand for woodland ES |  | LUC |
|  | Local (traditional and/or spiritual) arrangements for communal land access breakdown leading to open access and thence overhavesting | (Matose & Wily 1996; Lorbach et al. 1996) | Open access to ES can lead to overuse |
|  | As woodland becomes scarce, access and use may become restricted / privatised | (Clarke et al. 1996) | Restricted access to ES |
|  | Scarcity drives enhanced management and conservation | (Fairhead & Leach 1995) | Changed access to ES, LUC |
|  | Market for carbon creates new land use incentives | (Wertz-kanounnikoff & Sitoe 2011) | LUC |
| Global and regional environmental change | Rising pCO_2_ | (Higgins & Scheiter 2012) | woodland ecology via increasing tree cover and reducing grass cover; potentially changing species composition |
|  | Rising temp | (Schlenker & Lobell 2010; Pienaar et al. 2015) | LUC (Reduced maize yields), altered woodland ecology, range shifts and species turnover |
|  | Changes in Precipitation | (Seth et al. 2013) | woodland ecology, soil erosion |
|  | Change in ENSO – increased frequency of mega droughts | (Cai et al. 2014; Davey et al. 2014) | Agric change |
|  | Increased N deposition | (Lamarque et al. 2013) | woodland ecology |
|  | Altered fire regimes | (Andela & van der Werf 2014) | Woodland ecology |

## References for Table S4

Alcamo, J. et al., 2005. Changes in Ecosystem Services and Their Drivers across the Scenarios. *Ecosystems and Human Well-being: Scenarios, Volume 2*, pp.297–373.

Andela, N. & van der Werf, G.R., 2014. Recent trends in African fires driven by cropland expansion and El Niño to La Niña transition. *Nature Climate Change*, 4(9), pp.791–795.

Anseeuw, W. et al., 2013. Creating a public tool to assess and promote transparency in global land deals: the experience of the Land Matrix. *Journal of Peasant Studies*, 40(3), pp.521–530.

Cai, W. et al., 2014. Increasing frequency of extreme El Niño events due to greenhouse warming. *Nature Climate Change*, 5(2), pp.1–6.

Clarke, J., Cavendish, W. & Coote, C., 1996. Rural households and miombo woodlands: use, value and management. In *The Miombo in transition: Woodlands and welfare in Africa*.

Davey, M.K., Brookshaw, A. & Ineson, S., 2014. The probability of the impact of ENSO on precipitation and near-surface temperature. *Climate Risk Management*, 1, pp.5–24.

Fairhead, J. & Leach, M., 1995. False forest history, complicit social analysis: Rethinking some West African environmental narratives. *World Development*, 23(6), pp.1023–1035.

Gasparri, N.I. et al., 2016. The Emerging Soybean Production Frontier in Southern Africa: Conservation Challenges and the Role of South-South Telecouplings. *Conservation Letters*, 9(1), pp.21–31.

Higgins, S.I. & Scheiter, S., 2012. Atmospheric CO2 forces abrupt vegetation shifts locally, but not globally. *Nature*, 488(7410), pp.209–212.

Lamarque, J.F. et al., 2013. Multi-model mean nitrogen and sulfur deposition from the atmospheric chemistry and climate model intercomparison project (ACCMIP): Evaluation of historical and projected future changes. *Atmospheric Chemistry and Physics*, 13(16), pp.7997–8018.

Laurance, W.F. et al., 2014. A global strategy for road building. *Nature*, 513(7517), pp.229–232.

Lorbach, I. et al., 1996. Strategies for local forest and woodland management in northern Mozambique. In *Sustainable management of indigenous forests in the dry tropics: Proceedings of an International Conference, Kadoma, Zimbabwe, 28 May - 1 June, 1996.* Forestry Commission, Zimbabwe, pp. 97–108.

Matose, F. & Wily, L., 1996. Institutional arrangements governing the use and the management of miombo woodlands. In B. M. Campbell, ed. *The Miombo in transition: Woodlands and welfare in Africa*. Center for International Forestry Research, pp. 195–220.

Messerli, P. et al., 2014. The geography of large-scale land acquisitions: Analysing socio-ecological patterns of target contexts in the global South. *Applied Geography*, 53, pp.449–459.

Mwampamba, T.H., 2007. Has the woodfuel crisis returned? Urban charcoal consumption in Tanzania and its implications to present and future forest availability. *Energy Policy*, 35(8), pp.4221–4234.

Owen, M., van der Plas, R.J. & Sepp, S., 2013. Can there be energy policy in Sub-Saharan Africa without biomass? *Energy for Sustainable Development*, 17(2), pp.146–152.

Pienaar, B. et al., 2015. Evidence for climate-induced range shift in Brachystegia (miombo) woodland. *South African Journal of Science*, 111(7-8), pp.1–9.

Pingali, P., Binswanger, H.P. & Bigot, Y., 1988. *Agricultural mechanization and the evolution of farming systems in sub-Saharan Africa*,

Rudel, T.K., 2013. The national determinants of deforestation in sub-Saharan Africa. *Philosophical transactions of the Royal Society of London. Series B, Biological sciences*, 368, p.20120405.

Sander, K., Gros, C. & Peter, C., 2013. Enabling reforms: Analyzing the political economy of the charcoal sector in Tanzania. *Energy for Sustainable Development*, 17(2), pp.116–126.

Schlenker, W. & Lobell, D.B., 2010. Robust negative impacts of climate change on African agriculture. *Environmental Research Letters*, 5(1), p.014010.

Seth, A. et al., 2013. CMIP5 projected changes in the annual cycle of precipitation in monsoon regions. *Journal of Climate*, 26(19), pp.7328–7351.

Shackleton, S. & Gumbo, D., 2010. Contribution of Non-wood Forest Products to Livelihoods and Poverty Alleviation. In E. N. Chidumayo & D. J. Gumbo, eds. *The Dry Forests and Woodlands of Africa Managing for Products and Services*. Earthscan, pp. 63–92.

UNPD, 2014. *World urbanization prospects: the 2014 revision, custom data acquired via website*,

van Vliet, N. et al., 2012. Trends, drivers and impacts of changes in swidden cultivation in tropical forest-agriculture frontiers: A global assessment. *Global Environmental Change*, 22(2), pp.418–429.

Wertz-kanounnikoff, S. & Sitoe, A., 2011. *How is REDD + unfolding in southern Africa’s dry forests ?*,

Zulu, L.C., 2010. The forbidden fuel: Charcoal, urban woodfuel demand and supply dynamics, community forest management and woodfuel policy in Malawi. *Energy Policy*, 38(7), pp.3717–3730.

Figure S5: a) Total area of large scale land acquisitions from 2000-2015. b) Number of large scale land acquisitions through time. Data from www.landmatrix.org (Anseeuw et al., 2013).

Anseeuw, W. et al., 2013. Creating a public tool to assess and promote transparency in global land deals: the experience of the Land Matrix. *Journal of Peasant Studies*, 40(3), pp.521–530.
